# Assessment of a Biosafety Device to Control Contamination by Airborne Transmission during Orthodontic/Dental Procedures

**DOI:** 10.1155/2022/8302826

**Published:** 2022-04-15

**Authors:** Josilene Santa Rita de Assis, Aguinaldo Silva Garcez, Hideo Suzuki, Victor Angelo Martins Montalli, Denise Nami Fujii, Michelle Bertini Prouvot, Selly Sayuri Suzuki

**Affiliations:** ^1^Department of Post-graduation in Orthodontics, Faculdade Sa˜o Leopoldo Mandic, Campinas, SP, Brazil; ^2^Department of Oral Microbiology, Division of Oral Medicine, Faculdade Sa˜o Leopoldo Mandic, Campinas, Sa˜o Paulo, Brazil

## Abstract

During the COVID-19 pandemic, dental professionals have faced high risk of airborne contamination between dentists, staff, and patients. The objective of this study was to evaluate the effect of an individual biosafety capsule in dentistry (IBCD) on reducing the dispersion of droplets and aerosols during orthodontic treatment and evaluate the clinician and patient's perception of using the IBCD. For the *in-vitro* part of the study, aerosol quantification was performed with and without the IBCD, using a nonpathogenic bacterial strain and viral strain in the reservoir and high-speed dental handpiece. Petri dishes with MRS agar were positioned from the head of the equipment at distances of 0.5, 1, and 1.5 m. After 15 minutes of passive aerosol sampling, the dishes were closed and incubated using standard aerobic conditions at 37°C for 48 hours to count colony forming units (CFUs). For the clinical part of the study, a questionnaire was sent to clinicians and patients to understand their perception of orthodontically treat and receive treatment using the barrier. The use of IBCD showed an effective means to reduce the dispersion of bacterial and viral contamination around 99% and 96%, respectively, around the main source of aerosol (*p* < 0.05). Clinical results showed a 97% bacterial reduction during patient's consultations (*p* < 0.05). The vast majority of clinicians and patients understand the importance of controlling the airborne dispersion to avoid contamination.

## 1. Introduction

As some countries mitigate lockdown and quarantine measures due to COVID-19 pandemic, returning to work has become a possibility for dentists as well as students at dental schools. To become a reality, it is imperative and urgent for clinicians to review biosafety protocols during the dental procedures. Center for Disease Control and Prevention (CDC) and American Dental Association (ADA) have launched guides with recommendations to be adopted prior, during, and after the patient consultation for both dentists and dental healthcare personnel (DHCP) [1,2].

Among occupations, dentists are considered the highest risk group of healthcare workers at risk for contracting infections in general, including COVID-19 (CDC, 2020) [3]. A recent study showed that there were a 5% higher incidence of COVID-19 cases among oral health professionals compared to general population [4,5]. SARS-CoV-2 RNA was found in gingival crevicular fluid and saliva [6] in asymptomatic as well as mildly symptomatic patients who tested positive for COVID-19 through PCR [7]. This finding incites that the oral cavity probably actively participates in SARS-CoV-2 transmission [8]. Therefore, measurements should be implemented to reduce contamination during aerosol-generating procedures (CDC, 2020) in order to safely treat even SARS-CoV-2 asymptomatic patients, as shown in this recent study [9].

Contamination is likely to be due to the nature of the dental practice, generating droplets and aerosols by high- and low-speed handpiece, and the close proximity between clinicians and patients during dental care [10].

A general recommendation should be implemented at the dental clinic before and after the patient's consultation such as the use of hand sanitizer, close contact avoidance (e.g., handshaking), and disinfection of all touched objects and surfaces at all times. Additionally, the use of proper personal protective equipment (PPE), such as N95 or FFP2 masks, gloves, gowns, and goggles or face shields, is endorsed to protect patients and DHCP from potentially infected environment [11]. As the entire world faces this pandemic, several Health and Dental Associations are preparing protocols to guide the management of clinical dental practices. Main recommendations from CDC to minimize contamination at dental clinics include avoiding aerosol-generating procedures whenever possible. If these procedures are necessary for dental care, use four-handed dentistry, high evacuation suction, and dental dams to minimize droplet splatter and aerosols [12]. Furthermore, Er:YAG laser, together with high-volume evacuators, used for the removal of caries produced the lowest aerosol amount compared to conventional dental handpieces, therefore improving biological safety at dental offices by reducing the risk of viral or bacterial transmission [5,13].

The transmission of SARS-CoV-2 virus is known to occur throughout droplets and/or aerosol originating from the oropharynx or by contacting with surfaces contaminated with the virus. CDC recommends that aerosol-generating procedures performed on known or suspected COVID-19 patients should be in negative pressure/airborne infection isolation room [14]. However, many patients at dental clinics may be asymptomatic or still nondiagnostic for COVID-9. Therefore, creating measurements to diminish aerosol exposure in the dental operatory room is crucial. The purpose of this study is to evaluate the use of a protective barrier to be used in orthodontic practice during routine orthodontic procedures and evaluate its effectiveness in controlling aerosol spreading.

## 2. Methods

**Figure 1:**
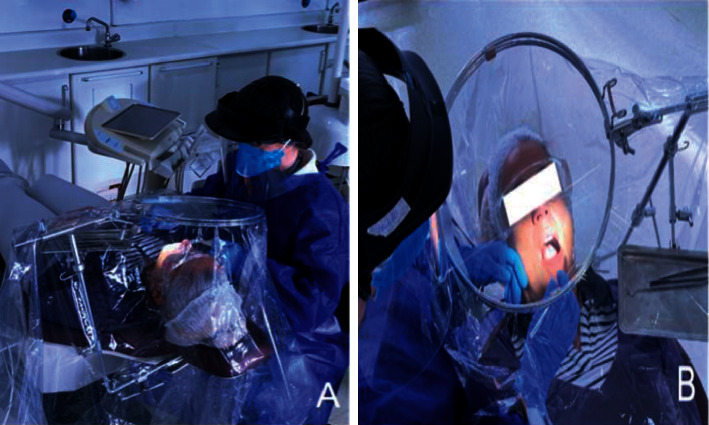
a and b) show the individual biosafety capsule device (IBCD) to the dental chair. The overhead ring can be adjusted to be rotated in all 3 dimensions, pitch, roll, and yaw in order to provide best visualization as well as freedom on hand and arm movements during procedures.

The new individual biosafety capsule device (IBCD) consists of stainless steel structures fixed at the base of the head support on the dental chair, including an overhead ring that holds a stretched PVC (polyvinyl chloride) plastic film sheet that covers the entire metal structure. PVC sheet used in this study measured 2 m length, 1.4 m width (approx. 78 × 55 inch), and a thickness of 20 microns. This plastic is commonly used for food wrapping. Although, PVC plastic film provides high transparency, it was heated using a hairdryer since it is a shrinkable material. The heating eliminated all wrinkles in the plastic and allowed a clear view. Additionally, vertical cuts allowing an opening for the hands were done using a silver tape to avoid plastic lifting which prevented airborne transmission to be escaped, the PVC film to be ripped, and facilitated visualization of the opening spaces during orthodontic procedures (Figure 1).

This study was approved by the Ethics Committee of Faculdade São Leopoldo Mandic, process number: ^#^4.412.029.

For bacterial strain and growth conditions, a nonpathogenic bacterial strain was used during droplet and aerosol production by a high-speed dental handpiece, to simulate a dental treatment, such a fixed appliance removal procedure.

In order to do that, a bottle of fermented milk probiotic drink Yakult® (supplied by Yakult Brazil Ltd.,), containing a single probiotic strain of *Lactobacillus casei* Shirota, was used to control the microbial load [15]. Viability tests were performed on three randomly selected bottles from separate batches to confirm a high viable count of 1.5 × 10^8^ (±1.1 × 10^3^) cells per bottle.

To produce the aerosol with the bacteriophage, a suspension of 10^5^ pfu/ml was prepared in sterile saline solution. In order to access small particles in the aerosol contamination, a bacteriophage for *Escherichia coli* previously isolated from domestic sewage samples was used [16]. The choice of a bacteriophage as a contamination model is due to its similar size to SARS-CoV-2 [17] and because this microorganism is harmless to humans.

**Figure 2:**
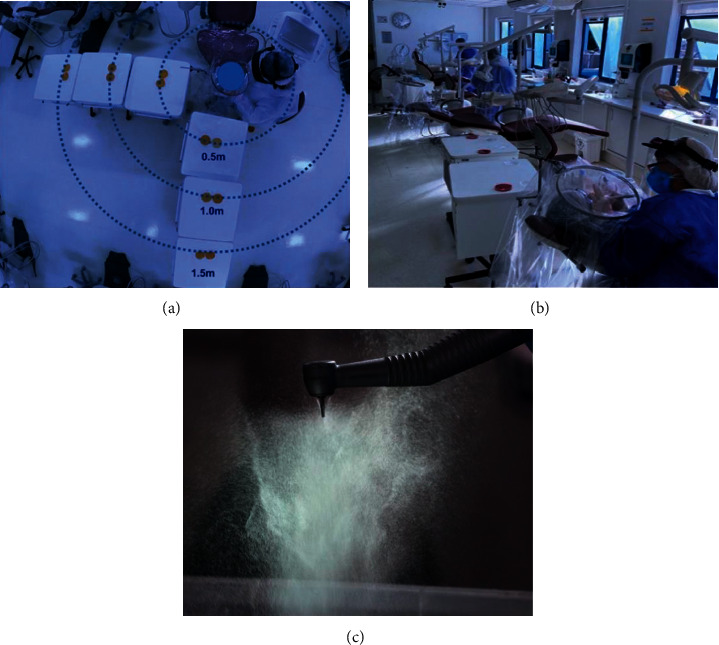
Petri dish distribution in relation to the head of the dental chair (a), Petri dishes at the orthodontic dental clinic during patient consultation with the IBCD (b) and high-speed dental handpiece in operation mode showing aerosol production (c).

To generate airborne transmission, a bottle of Yakult (80 ml) was added to the water reservoir of a dental chair filed phosphate-buffered saline (PBS) or the reservoir was filled with 400 ml of phage suspension. From the dental air compressor, air pressure is delivered to the dental chair. A high-speed air turbine handpiece (300,000 rpm) (605C-Kavo, Brazil) with a diamond tip ^#^2130 (FGM, Brazil) connected to the tip was turned on for 1 minute over a dental model, simulating the removal of resin remaining after fixed appliance debonding in one tooth crown (Figure 2). This procedure generated splatter, droplets, and aerosols in the environment from the fluid reservoir containing bacteria and virus.

Bacterial or viral contamination in the air was measured by passive sampling, to measure the rate at which viable particles settle on surfaces [18].

The quantification of airborne transmission was performed with and without the IBCD. Petri dishes with MRS agar (Man, Rogosa Sharpe agar – Neogen, USA) for bacteria or soft TSA (Triptone soya agar, Kasvi, Brazil) incubated with *E. coli* (ATCC 25922) for virus were positioned from the headboard of the equipment at distances of 0.5, 1, and, 1.5 m, at 90 and 0-degree angles and also on the floor and ceiling (Figure 2). Headrest from the dental chair where high-speed handpiece was operated (source of contamination) and auxiliary tables containing the Petri dishes were positioned at the same height, 80 cm from the floor. Petri plates were also placed 1.70 m away for the ceiling and 80 cm from the floor, and directly above and below the headrest. For passive bacterial sampling, Petri dishes were kept open for 30 minutes [19]. An additional Petri dish was positioned inside the IBCD to evaluate the contained airborne transmission inside the device. The same procedure was done 3 times on different days for both groups.

In order to avoid drafts, doors and windows were kept closed during the procedures and the air conditioner was turned off.

After 15 minutes of particles' passive sampling, Petri dishes were closed and incubated using standard aerobic conditions at 37°C for 24 h for colony-forming unit (CFU) or plaque-forming unit (PFU) counting. The mean values from the samples in each group were then computed.

The degree of airborne transmission during orthodontic procedures between two open setup postgraduation clinics was compared, including 12 dental chairs (6 in use for distance purposes during pandemic), with and without the use of IBCD by orthodontic students. An adapted model of a passive viable surface settling was used using plates containing chocolate agar or blood agar (Biomérieux, São Paulo—Brazil), a nonselective, enriched growth medium used for bacterial sampling. Three plates were left open in the center of each clinic during 4 hours while students treated patients. An average of 20 patients were seen in each clinic (Figure 2).

After 6 months of adopting the IBCD, a questionnaire was sent to clinicians and patients in order to better understand their perception of orthodontically treat and receive treatment using the barrier. Questions were sent to clinicians and patients using Google forms when they were not identified, and all agreed to participate in the study. A total of 50 clinicians, including students enrolled in the second and third years of the Orthodontic program, orthodontists with different years of experience and faculty members, and 40 adult patients (mean age 28.3 ± 7.1) were included. Sample description is represented in Table 1.

Clinician's experience during the use of the IBCD regarding the level of discomfort, adaptation, difficulty on using the device while performing orthodontic procedures, opinion on aerosol controlling and level of safety, the possibility of recommending to a colleague and procedure performance was assessed using a questionnaire including closed questions with 5 statements each. The patient's experience during the consultation in the IBCD was assessed by questions about the level of safety, claustrophobia, discomfort, and satisfaction, as well as their perception of the IBCD regarding the importance of avoiding contamination, possibility of recommending to others, and concern of receiving dental treatment during the pandemic situation.

Data were examined for normality by the Shapiro–Wilk test. As data demonstrated normality, all analyses were then performed using parametric methods. The differences in CFU for the different distances were compared by two-way ANOVA, followed by Tukey's test. To analyze clinicians' and patients' experience with the IBCD, chi-squared tests were performed comparing the response of every question. The level of significance was established at 5%. All statistical analyses were performed with GraphPad Prism v8.0 (San Diego, CA, USA).

## 3. Results and Discussion

### 3.1. Assessment of Airborne Transmission Control

**Figure 3:**
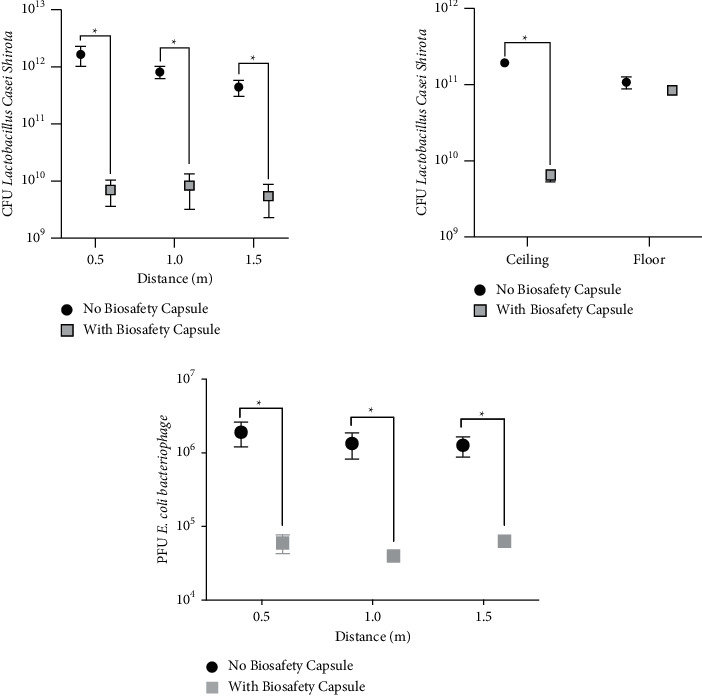
Aerosol production with and without the individual biosafety capsule device (IBCD) using *Lactobacillus* and *E. coli* bacteriophage.

Droplets and aerosols with or without the use of the barrier, as expected, was higher on 0.5 m, followed by 1 and 1.5 m. However, the IBCD reduced the deposition of bacteria over the Petri dishes almost to 98.86%, on average (Figure 3). The reduction was 99.5% for 0.5 m, 98.8% for 1 m, and 98.3% for 1.5 m (*p* < 0,05). Figure 3 also shows the results of viral contamination with and without the use of the barrier, as follows: 96.8% for 0.5 m, 97% for 1 m, and 95% for 1.5 m (*p* < 0.05).

Using the IBCD, the most contaminated sample was on the plate positioned inside the barrier (data not shown), showing the PVC sheet helps to keep droplets and aerosols inside the device, avoiding/reducing the dispersion to the environment.

At the ceiling, there was a reduction of 98% of the bacterial deposition with the use of the IBCD (*p* < 0.05); however, on the floor no significant difference was found between the two groups (Figure 3).

The risk of contamination by airborne transmission during dental treatment is high since, in dental procedures, especially while using a high-speed dental handpiece, ultrasound scaling or water spray syringe, generating droplets and aerosols disperse in all directions over a distance that could be as far as 2.0 m from the mouth of the patient[20], which corroborates with the results of this study and other studies which also suggested that the use of Er:YAG lasers during dental treatments (including orthodontic brackets' debonding) significantly reduces the amount of aerosol in the dental clinics compared to conventional high- and low-speed handpieces, especially during the COVID-19 pandemic [5,13].

**Figure 4:**
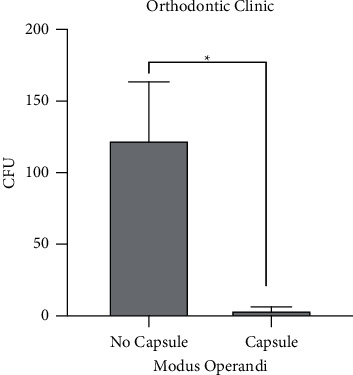
Comparison of aerosol production among two dental clinics with and without the IBCD.

When comparing contamination in two clinics with and without the use of the IBCD, the results showed that the barrier was able to reduce air contamination derived by orthodontic procedures during patient's consultation by 97% compared to its nonuse (*p* < 0.05) (Figure 4).

During an orthodontic clinical procedure, equipment, materials, and professionals are commonly located in the surrounding areas within droplet and aerosol dispersion radius. Therefore, there is a high risk of cross-contamination [21]. As far as dental care professionals, the COVID-19 pandemic brought to life the need to reinforce personal protective equipment—eyewear, respirator, and face shield—to prevent contamination. Moreover, the implementation of effective infection control by rinsing antimicrobial mouthwash solutions combined with preoperative brushing is indicated to reduce the microbial load, as well as its spread [22,23]. Ventilation in the room is recommended to dilute the viral load, either by mechanical or natural ventilation, creating a draught inside the room [24]. However, control of aerosol is still imperative to reduce airborne contamination.

To measure the contamination in a clinical environment, a strain of harmless bacteria and bacteriophage chosen were 3 *μ*m and 0.1 *μ*m, respectively. The latest one presenting similar size than SARS-CoV-2 virus. During dental procedures, several equipment are used generating a large amount of splatters, droplets, and aerosols, such as ultrasonic scaling, triplice syring, high-speed and low-speed handpieces, among others [25]. Five microns has been used historically to distinguish aerosols from droplets [26,27]; a recent article has suggested that the size distinction between aerosols and droplets should be 100 *μ*m, since this particle size that can remain suspended in still air for more than 5 s from a height of 1.5 m typically reach a distance of 1 to 2 m from the source, and also can be inhaled. The same study has reported that particles as small as 5 *μ*m takes little more than 30 minutes to settle to the ground from 1.5 m height [28]. Therefore, our study was conducted in an indoor environment and passive sampling was conducted during 30 mins. Since microorganisms are attached to one of these vehicles (droplets and aerosols), controlling contamination in the dental office, between clinicians, staff, and patients, has always been a major concern during dental treatment, with much greater attention since COVID-19 outbreak.

The results of this study showed that the use of IBCD is an effective method to reduce air contamination in more than 98.8% bacterial and 96.2% for viral contamination around the main source of airborne transmission. Another recent study has found similar results, with a reduction of 96% of bacterial load using an individual biosafety barrier in a simulation using the high-speed drill and concluding that the proposed device is also a viable option to prevent bioaerosol dispersion in the dental environment [15]. Other measurements may also be recommended to reduce contamination such as the use of ultrasound device, high-volume evacuator, saliva ejector with extraoral vacuum, and hand instrumentation when possible [24].

Although clinical results during dental procedures in patients showed 97% of bacterial reduction using the IBCD, the present study used a passive sampling to measure bacterial and viral contamination in a dental setting-controlled simulation with and without the presence of a barrier during a period of 30 minutes. A more active methodology in a longer period of time, measuring smaller particles suspended in the air is recommended in order to precisely understand the effectiveness of the IBCD on air contamination.

### 3.2. Clinician and Patient's Perception of Using the Individual Biosafety Capsule Device (IBCD)

Regarding the clinician's experience, all questions showed significant differences between the given statements (*p* < 0.05). The majority of the dentists considered the IBCD either comfortable or very comfortable to work with, totalizing 60% of them. On the other hand, 32% of the clinicians answered as uncomfortable, on which most of them were orthodontic enrolled students. Most of clinicians were adapted and well-adapted to the IBCD (total of 82%) and consider working with the barrier easy or very easy (66%). As for the level of importance for droplet/aerosol control, 98% considered the use of the barrier as important and very important and 92% felt safe and very safe working with it.

Similarly, all patient's questions also showed significant differences between the statements (*p* < 0.05). All patients (100%) answered that they felt protected or very protected under the IBCD, 85% stated as comfortable and very comfortable, and the level of claustrophobia was 92.5% as very little or no claustrophobic at all. As the importance level of using the device to avoid contamination, patients' answers were 100% important and very important and 92.5% would recommend it to others. Regarding patient's concern of receiving orthodontic treatment during the current pandemic situation, 92.5% are concerned and very concerned (Table 2).

Orthodontic programs of dental schools in Brazil, as well as private practice orthodontists, have embraced the IBCD a standardized biosafety protocol to see patients for orthodontic consultation in the past 6 months. Both clinicians' and patients' response has been positive towards the use of the barrier as observed on the questionnaire. Patients mention feeling safer and protected by the barrier since it isolates them from the dentist, staff, and surroundings, allowing them to feel more comfortable opening their mouth to receive orthodontic treatment. They perceived it as a gesture of care, especially when children are seen, increasing their level of satisfaction and are more likely to recommend to other patients. Initially, a polypropylene material (TNT) was used over the structure of the IBCD (except for the overhead ring's field of view) but patients reported claustrophobic feelings compared to the full plastic barrier.

The results of the questionnaire showed that the vast majority of clinicians and patients understand the importance of controlling the airborne dispersion to avoid contamination. Although not all clinicians found comfortable working with the IBCD compared to not having it (32%), it seems the most of them were capable to adapt to it (92%), in an easy or very easy manner (66%). The reason might be that they feel safe (92%) and that most orthodontic procedures can be performed using the IBCD (90%), except for taking photos.

Limitations to this device may include the use of plastic which is not environmentally sustainable in a long term. In addition, most importantly, the use of this device does not replace any recommendations by governmental agencies while seeing patients during the COVID-19 pandemic. Also, although this study showed a significant bacterial load reduction and aerosol contention, additional precautions should be also implemented such as the use of high-pressure saliva ejectors and UV-C light for decontamination to avoid SARS-CoV-2 transmission.

A simple and affordable device was developed with the potential to reduce airborne transmission dispersion during dental treatment. Measurements of air contamination confirm the reduction of bacterial load on air during and after the biosafety barrier usage. The use of this device may diminish aerosol transmission, serving as a protection against sneeze, cough, and speech; it is well accepted by patients, does not interfere with orthodontic procedures, and represents an affordable and simple device. Reducing aerosol transmission may play an important role in decreasing exposure to other airborne pathogens to the dentist and staff.

## 4. Conclusions

The results of this study showed that the use of the biosafety device is an effective means to reduce air contamination by more than 99% of bacterial contamination around the main droplet/aerosol source. The use of this device can decrease aerosol transmission, serves as protection against sneezing and/or coughing, and is well accepted by patients and clinicians.

## Figures and Tables

**Table 1:** Sample characterization of the clinicians and patients who answered the Google forms' questionnaires.

Dentists				Patients			
Sample distribution	Student	20	Total *n* = 50	Appliance	Fixed appliance	30	Total *n* = 40
	Faculty	10			Aligners	10	
	Clinician—less than 5 years of ortho experience	6		Treatment duration	Less than 1 year	6	
	Clinician—6–10 years of ortho experience	7			Less than 2 years	12	
	Clinician—more than 10 years of ortho experience	7			Less than 3 years	20	
					More than 3 years	2	

**Table 2:** Results of dentists' and patients' experience using the individual biosafety capsule device (IBCD).

Dentists				Patients			
		*n*	%		*n*	%	
Level of discomfort with the IBCD	Very uncomfortable	0 (0%)	*X* ^2^ = 34.0 DF = 4 *P* < 0.0001^*∗*^	Level of protection with the IBCD	Not protected	0 (0%)	*X* ^2^ = 75.0 DF = 4 *P* < 0.0001^*∗*^
	Uncomfortable	16 (32%)			Somewhat protected	0 (0%)	
	Indifferent	4 (8%)			Indifferent	0 (0%)	
	Comfortable	20 (40%)			Protected	20 (50%)	
	Very comfortable	10 (20%)			Very protected	20 (50%)	
Level of adaptation with the IBCD	Not adapted	0 (0%)	*X* ^2^ = 113.3 DF = 4 *P* < 0.0001^*∗*^	Level of claustrophobia with the IBCD	Very claustrophobic	0 (0%)	*X* ^2^= 123.4 DF = 4 *P* < 0.0001^*∗*^
	Somewhat adapted	3 (6%)			Reasonably claustrophobic	2 (5%)	
	Indifferent	1 (2%)			Indifferent	1 (2.5%)	
	Adapted	36 (72%)			Very little claustrophobic	4 (10%)	
	Very adapted	10 (20%)			Not claustrophobic	33 (82.5%)	
Level of difficulty on working with the IBCD	Very hard	0 (0%)	*X* ^2^ = 46.2 DF = 4 *P* < 0.0001^*∗*^	Level of discomfort with the IBCD	Very uncomfortable	2 (5%)	*X* ^2^ = 47.5 DF = 4 *P* < 0.0001^*∗*^
	Hard	9 (18%)			Uncomfortable	1 (2.5%)	
	Indifferent	8 (16%)			Indifferent	3 (7.5%)	
	Easy	26 (52%)			Comfortable	13 (32.5%)	
	Very easy	7 (14%)			Very comfortable	21 (52.5%)	
Importance of aerosol control	Not important	0 (0%)	*X* ^2^ = 126.8 DF = 4 *P* < 0.0001^*∗*^	Level of satisfaction with the IBCD	Very unsatisfied	0 (0%)	*X* ^2^ = 41.9 DF = 4 *P* < 0.0001^*∗*^
	Somewhat important	0 (0%)			Unsatisfied	1 (2.5%)	
	Indifferent	1 (2%)			Indifferent	1 (2.5%)	
	Important	12 (24%)			Satisfied	14 (35%)	
	Very important	37 (74%)			Very satisfied	14 (35%)	
Level of safety using the IBCD	Very unsafe	0 (0%)	*X* ^2^ = 75.5 DF = 4 *P* < 0.0001^*∗*^	Importance of avoiding contamination	Not important	0 (0%)	*X* ^2^ = 128.8 DF = 4 *P* < 0.0001^*∗*^
	Unsafe	0 (0%)			Somewhat important	0 (0%)	
	Indifferent	4 (8%)			Indifferent	0 (0%)	
	Safe	19 (38%)			Important	7 (17.5%)	
	Very safe	27 (54%)			Very important	33 (82.5%)	
Possibility on recommending IBCD to others	Not likely	0 (0%)	*X* ^2^ = 63.6 DF = 4 *P* < 0.0001^*∗*^	Possibility of recommending IBCD to others	Not likely	0 (0%)	*X* ^2^ = 85.9 DF = 4 *P* < 0.0001^*∗*^
	Unlikely	4 (8%)			Unlikely	2 (5%)	
	Indifferent	2 (4%)			Indifferent	1 (2.5%)	
	Likely	21 (42%)			Likely	9 (22.5%)	
	Very likely	24 (48%)			Very likely	28 (70%)	
Capable of performing all procedures with the IBCD?	Yes	43 (86%)	*X* ^2^ = 51.8 DF = 1 *P* < 0.0001^*∗*^	Level of concern on attending orthodontic consultation	Very unconcerned	0 (0%)	*X* ^2^ = 88.79 DF = 4 *P* < 0.0001^*∗*^
	No	7 (14%)			Unconcerned	0 (0%)	
	Comments: Photographs (*n* = 5), surgery (*n* = 2)				Indifferent	3 (7.5%)	
					Concerned	8 (20%)	
					Very concerned	29 (72.55%)	

^*∗*^level of significance *p* < 0.05.

## Data Availability

All data used to support the findings of this study are available from the corresponding author upon request.
